# Performance of the BioFire FilmArray Pneumonia Panel Plus Compared to Standard Microbiology in Lung Transplant Donor and Recipient Samples: A Prospective Cohort Study

**DOI:** 10.1111/tid.70186

**Published:** 2026-02-20

**Authors:** Andrea Lombardi, Giulia Renisi, Lorenzo Rosso, Letizia Morlacchi, Jacopo Fumagalli, Lisa Cariani, Ilaria Righi, Valeria Rossetti, Arianna Liparoti, Cecilia Azzarà, Davide Mangioni, Laura Alagna, Paola Saltini, Maria Francesca Liporace, Chiara Abbruzzese, Annapaola Callegaro, Francesco Blasi, Giacomo Grasselli, Mario Nosotti, Alessandra Bandera

**Affiliations:** ^1^ SC Malattie Infettive Fondazione IRCCS Ospedale Maggiore Policlinico Milan Lombardy Italy; ^2^ Dipartimento Di Fisiopatologia Medico‐Chirurgica e Dei Trapianti Università degli Studi di Milano Milan Lombardy Italy; ^3^ SC Chirurgia Toracica e Trapianti di Polmone Fondazione IRCCS Ospedale Maggiore Policlinico Milan Lombardy Italy; ^4^ SC Pneumologia e Fibrosi Cistica Fondazione IRCCS Ospedale Maggiore Policlinico Milan Lombardy Italy; ^5^ SC Anestesia e Terapia Intensiva Adulti Fondazione IRCCS Ospedale Maggiore Policlinico Milan Lombardy Italy; ^6^ SC Microbiologia e Virologia Fondazione IRCCS Ospedale Maggiore Policlinico Milan Lombardy Italy

**Keywords:** donor‐derived infection, fast microbiology, lung transplantation, perioperative prophylaxis

## Abstract

**Background:**

Lung transplantation (LuTx) is hampered by infectious risks. Perioperative antibiotic prophylaxis (PAP) is widely used; however, real‐time adjustment is hindered by the timing of standard microbiology. Syndromic molecular panels offer rapid results, yet their integration into PAP strategies remains unclear.

**Methods:**

We conducted a prospective cohort study comparing the BioFire FilmArray Pneumonia Panel Plus (PN*plus*) with standard of care (SOC) on bronchoalveolar lavage (BAL) samples obtained from donors at procurement and from recipients 72 h after LuTx. Concordance between PN*plus* and SOC was assessed for bacterial species and antimicrobial resistance genes.

**Results:**

Fifty‐three donor‐recipient pairs were analyzed. In donor BAL, PN*plus* identified at least one pathogen in 67.9% (36/53) of cases versus 63.5% (33/53) by SOC, with a markedly shorter time to result (221 min vs. 5.3 days). Concordance between PN*plus* and SOC for bacterial species was substantial (Cohen's *κ* = 0.654), particularly for *Staphylococcus aureus* (Cohen's *κ* = 0.689), *Streptococcus pneumoniae* (Cohen's *κ* = 0.658), and *Pseudomonas aeruginosa* (Cohen's *κ* = 0.731). In recipient BAL, PN*plus* detected pathogens in 61.5% (32/53) compared to 47.2% (25/53) with SOC, but overall concordance was only moderate (*κ* = 0.365). Resistance gene concordance was minimal, with PN*plus* often identifying additional determinants not confirmed by SOC. Viruses were detected exclusively by PN*plus*, while fungi were identified only by SOC.

**Conclusion:**

PN*plus* provides rapid, clinically relevant pathogen detection in LuTx, showing substantial agreement with SOC in donor samples and offering potential to support PAP adjustment. In early post‐transplant recipient BAL, interpretation requires caution, and SOC remains indispensable, particularly for detecting fungi and confirming phenotypic resistance.

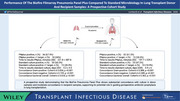

## Graphical abstract

A prospective study demonstrating that the BioFire Pneumonia Panel Plus shows substantial concordance with culture in donor samples and moderate concordance in recipient samples, supporting its potential role in guiding perioperative antibiotic prophylaxis in lung transplantation.

**Abbreviations**
BAL: bronchoalveolar lavage
CF: cystic fibrosis
CLAD: chronic lung allograft dysfunction
COPD: chronic obstructive pulmonary disease
DDI: donor‐derived infection
DTR: difficult‐to‐treat resistance
ESBL: extended‐spectrum beta‐lactamase
ILD: interstitial lung disease
LUTR: LuTx recipients
LuTx: lung transplantation
PAP: perioperative antibiotic prophylaxis
PN*plus*
: BioFire FilmArray Pneumonia Panel Plus
SOC: standard of care

## 1 Introduction

Lung transplantation (LuTx) represents a potentially curative procedure for end‐stage lung diseases, but infectious complications still hamper its short and long‐term outcomes. The procedure is characterized by the risk of developing surgical‐site infections, caused by pathogens that are carried by the donor (donor‐derived infections [DDI]), those that can be transferred or amplified by potential perfusion procedures of the organ, and those that are part of the skin and respiratory tract flora of the recipient.

Perioperative antibiotic prophylaxis (PAP) is administered to prevent these infections. However, the global panorama is quite varied, with a multitude of different antibiotics administered, frequently in combination, and with different durations, especially when the recipient has a chronic respiratory tract infection/colonization by a microorganism with a difficult‐to‐treat profile or carrying antimicrobial resistance genes [1, 2].

The combination of a syndromic molecular panel and standard culture (SOC) has been advocated as a potential tool to guide and adjust PAP in LuTx recipients (LUTR) [3]. Syndromic molecular panels enable the simultaneous detection of multiple targets, allowing for the screening of predefined infectious disease pathogens and resistance mechanisms associated with a specific clinical syndrome within a 1‐h timeframe. This creates opportunities for rapid diagnostics and intervention [4].

However, the evidence concerning syndromic molecular panels in the context of PAP, and more generally, LuTx, is limited [5, 6, 7, 8, 9, 10]. Overall, considering all the studies performed among LUTR, the syndromic panels provide results in a significantly shorter time compared to SOC, with an agreement between the two approaches ranging between 0.56 and 0.98. The reported sensitivity and specificity of syndromic panels range from 58% to 94% and from 78% to 100%, respectively. However, many microorganisms, especially *Haemophilus parainfluenzae*, *Candida* spp., and *Aspergillus* spp., are not detected by the syndromic panels, mainly because these microbes are not among the targets included in these instruments [11].

Therefore, a crucial step in defining the possibility of implementing syndromic molecular panels as a tool to support PAP in LuTx is to verify the performance of this instrument in the specific setting of the perioperative scenario and its concordance with SOC. To address this question, we compared the results of the syndromic molecular panel and SOC, performed on bronchoalveolar lavage (BAL) samples collected from the donor at procurement and from the recipient 72 h after LuTx, in a prospective cohort of LUTR.

## 2 Methods

The PneumoArray study is a prospective investigation comparing the syndromic molecular panel BioFire Filmarray Pneumonia Panel Plus (PN*plus*) with traditional culture‐based methods for the microbiological diagnosis of BAL samples from LuTx donors and recipients. The study included all the consecutive LuTx performed at the LuTx Centre of the Fondazione IRCCS Ca' Granda Ospedale Maggiore Policlinico of Milan, Italy, in the period 01/02/2023‐31/12/2024.

For each patient, demographic and clinical data were extracted from electronic records.

The PN*plus* is a multiplex PCR assay detecting 15 common bacterial pathogens, three atypical bacteria, eight respiratory viruses, and seven genetic resistance determinants. Resistance genes detected are reported only when a potential carrier bacterium is present. Common bacteria are reported semi‐quantitatively (10^4^→10^7^ genomic copies/mL), while atypical bacteria, viruses, and resistance determinants are qualitatively (detected / not detected). The list of targets identified by PN*plus* is provided in Table S1.

Microbiological diagnostics were performed in the respective routine laboratories according to national standards. All BAL samples tested with the PN*plus* panel were also analyzed by conventional culture. Specifically, 90 µL of BAL fluid was inoculated on Columbia agar with 5% sheep blood (COS), CNA agar, *Haemophilus* chocolate agar (HAE2), MacConkey agar (MCK), and Sabouraud Gentamicin Chloramphenicol agar (SGC2) (bioMérieux, France).

Plates were incubated at 35°C for 48 h under standard and CO_2_‐enriched conditions and examined daily. Bacterial identification was performed using MALDI‐TOF MS (VITEK MS PRIME, bioMérieux). Antimicrobial susceptibility testing (AST) was carried out by disk diffusion or Vitek‐2 system and interpreted according to EUCAST clinical breakpoints. Phenotypic resistance detected by SOC was mapped to corresponding resistance genes when applicable.

In our clinical practice, bronchoscopy through an endotracheal tube is routinely performed by the procurement team in all lung donors upon arrival at the donor hospital to evaluate the airway anatomy, to assess the presence of secretions and their characteristics, and to obtain samples for microbiological investigations. When evidence of bronchial secretions is present, a bronchial wash is performed rather than a targeted BAL. In these cases, secretions are gently aspirated from the airways using small aliquots of sterile isotonic saline to facilitate their removal and achieve complete bronchial toilet. When no secretions are present and the airways appear normal, a standard BAL is performed in the middle lobe, unless imaging or CT findings indicate a target area. The procedure is carried out using at least 350 mL of sterile saline instilled in sequential 20 mL aliquots, with gentle aspiration after each instillation. Samples are stored at room temperature when transport time to our center is less than 2 h; otherwise, they are kept in the refrigerator until processing.

In all lung transplant recipients, a bronchoscopy with BAL is performed at 72 h post‐transplant, as per the study protocol. The bronchoscope is wedged into a segmental/subsegmental bronchus of the middle lobe, in the absence of specific clinical or radiological indications for another site. At least 50 mL of sterile saline is instilled in sequential 20 mL aliquots, with immediate low‐pressure suctioning to prevent airway collapse and maximize fluid recovery. BAL samples are stored at room temperature for short‐term in the bronchoscopy area until transportation to the clinical laboratory.

This analysis presents the primary outcome of the PneumoArray study, which is to assess the microbiological concordance between molecular diagnostic and SOC on the donor's BAL lavage prior to LuTx. It also evaluates one of the secondary outcomes, the microbiological concordance between these methods on the recipient's BAL, performed 72 h after LuTx. Concordance was defined as the identification of the same bacterial species in PN*plus* and standard culture; concordance was not assessed for targets not included in the syndromic panel.

Continuous variables were described as median and interquartile range (IQR), and differences between groups were searched using the Mann–Whitney *U* test. Categorical variables were reported as counts and percentages; Pearson's chi‐square test was used to compare groups. All statistical tests were two‐tailed, and the significance threshold was *p *< 0.05. Analyses were performed with R.

Written informed consent was obtained from all participants, the study was approved by the local ethical committee (665_2022bis), registered in the ClinicalTrial.gov platform (NCT05960383), and conducted following the Helsinki Declaration.

## 3 Results

The study enrolled 60 LuTx candidates. Of these, seven were excluded because PN*plus* was not performed on both the donor's and the recipient's samples due to logistical constraints. Overall, 53 donor‐recipient couples are included in the current analysis (Figure S1).

Males constituted the majority (62.3%) of this cohort, with a mean age at LuTx of 52.7 years; interstitial lung disease and cystic fibrosis/bronchiectasis were the most common indications to transplantation, being the underlying lung disease in 15 and 12 patients, respectively. Double LuTx was carried out in most cases (88.7%), while donor donation after circulatory death occurred in ten instances. (Table 1)

**TABLE 1:** Clinical characteristics of lung transplant recipients at the time of transplantation.

*N*	53
Gender	
Male, *n* (%)	33 (62.3%)
Female, *n* (%)	20 (37.7%)
Age	52.7 ± 12.4
BMI, Kg/m^2^ (SD)	24.2 ± 4.2
Fraction of inspired O_2_ at rest pre‐transplant, (SD)	15.9 ± 14.3
FEV1, L (SD)	1.77 ± 3.60
Donor donation after circulatory death, *n* (%)	10 (18.9%)
DCD2, *n* (%)	5 (9.4%)
DCD3, *n* (%)	5 (9.4%)
Type of transplantation	
Single lung, *n* (%)	6 (11.3%)
Double lung, *n* (%)	47 (88.7%)
Transplant indication	
Cystic fibrosis and bronchiectasis, *n* (%)	12 (22.6%)
ILD, *n* (%)	15 (28.3%)
Connective tissue disease, *n* (%)	6 (11.3%)
COPD, *n* (%)	8 (15.1%)
Lymphangioleiomyomatosis and sarcoidosis, *n* (%)	2 (3.8%)
Other, *n* (%)	16 (30.2%)
Comorbidities	
Diabetes, *n* (%)	9 (17%)
Obesity, *n* (%)	3 (5.7%)
Chronic liver disease, *n* (%)	3 (5.7%)
Chronic kidney disease, *n* (%)	0
Chronic heart disease, *n* (%)	7 (13.2%)
Dialysis, *n* (%)	0
Charlson comorbidity index	
0, *n* (%)	1 (1.9%)
1, *n* (%)	10 (18.9%)
2, *n* (%)	13 (24.5%)
3, *n* (%)	20 (37.7%)
4, *n* (%)	7 (13.2%)
5, *n* (%)	2 (3.8%)

Abbreviations: BMI, body mass index; COPD, chronic obstructive pulmonary disease; FEV1, forced expiratory volume; ILD, interstitial lung disease.

On the donor's BAL sample, the PN*plus* detected at least one target in 36 (67.9%) patients, with an average time from sample acceptance in the microbiology laboratory to results availability of 221.8 min (SD 387.8). Conversely, SOC identified at least one microorganism in 33 patients (63.5%), with an average time from sample acceptance in the microbiology laboratory to definitive results of 5.3 days (SD 9.5). The microorganisms most frequently identified only by the PN*plus* were Coronavirus and Human Rhinovirus/Enterovirus, with three cases each. Conversely, *Citrobacter koseri*, *Enterobacter hormaechei*, *Aspergillus fumigatus*, and *Candida albicans* were the microorganisms most frequently identified only by SOC, with two cases each. Regarding antimicrobial resistance genes, the PN*plus* detected three targets: one VIM (Verona integron‐encoded metallo‐beta‐lactamase), one CTX‐M, and one mecA/mecC. Instead, no resistance genes were identified by SOC (Table 2).

**TABLE 2:** Microbiological results of the donor's (A) and recipient's (B) bronchoalveolar lavage sample, based on the Pneumonia *plus* (PN*plus*) panel and standard culture.

***N***	**53**
**(A)**	
PN*plus* positive, *n* (%)	36 (67.9%)
PN*plus* positive > 1 target, *n* (%)	18 (34%)
Time to results PN*plus*, minutes (SD)	221.8 ± 387.8
Standard culture positive, *n* (%)	33 (63.5%)
Standard culture positive > 1 target, *n* (%)	15 (28.3%)
Time to results standard culture, days (SD)	5.3 ± 9.5
*Aspergillus* spp. galactomannan antigen test positive, *n* (%)	1 (4%)
*Aspergillus* spp. DNA test positive, *n* (%)	0
Mycobacteria culture positive, *n* (%)	0
Microorganisms identified exclusively by PN*plus*, *n*	10
Coronavirus, *n* (%)	3 (30%)
Human Rhinovirus/Enterovirus, *n* (%)	3 (30%)
*Streptococcus agalactiae*, *n* (%)	2 (20%)
*Moraxella catarrhalis*, *n* (%)	1 (10%)
Influenza B, *n* (%)	1 (10%)
Resistance genes identified by PN*plus*, *n*	3
VIM	1 (33%)
CTX‐M	1 (33%)
mecA/mecC	1 (33%)
Microorganisms identified exclusively by standard culture, *n*	17
*Candida albicans*, *n* (%)	3 (7.7%)
*Citrobacter koseri*, *n* (%)	2 (11.8%)
*Enterobacter hormaechei*, *n* (%)	2 (11.8%)
*Aspergillus fumigatus*, *n* (%)	2 (11.8%)
*Burkholderia gladioli*, *n* (%)	1 (5.9%)
*Candida glabrata*, *n* (%)	1 (5.9%)
*Candida tropicalis*, *n* (%)	1 (5.9%)
*Fusarium proliferatum*, *n* (%)	1 (5.9%)
*Enterococcus faecalis*, *n* (%)	1 (5.9%)
*Stenotrophomonas maltophilia*, *n* (%)	1 (5.9%)
*Aeromonas hydrophila*, *n* (%)	1 (5.9%)
*Citrobacter werkmanii*, *n* (%)	1 (5.9%)
Resistance genes identified by standard culture, *n*	0
**(B)**	
PN*plus* positive, *n* (%)	32 (61.5%)
PN*plus* positive > 1 target, *n* (%)	15 (28.3%)
Time to results PN*plus*, minutes (SD)	157.7 ± 229.2
Standard culture positive, *n* (%)	25 (47.2%)
Standard culture positive > 1 target, *n* (%)	10 (18.9%)
Time to results standard culture, days (SD)	4.9 ± 6
*Aspergillus* spp. galactomannan antigen test positive, *n* (%)	7 (23.3%)
*Aspergillus* spp. DNA test positive, *n* (%)	1 (3.7%)
Mycobacteria culture positive, *n* (%)	0
Microorganisms identified exclusively by PN*plus*, *n*	21
Human Rhinovirus/Enterovirus, *n* (%)	7 (33.3%)
*Haemophilus influenzae*, *n* (%)	5 (23.8%)
Coronavirus, *n* (%)	3 (12.5%)
*Enterobacter cloacae*, *n* (%)	2 (8.3%)
Influenza B, *n* (%)	1 (4.7%)
*Streptococcus agalactiae*, *n* (%)	1 (4.7%)
*Streptococcus pneumoniae*, *n* (%)	1 (4.7%)
*Moraxella catarrhalis*, *n* (%)	1 (4.7%)
Resistance genes identified by PN*plus*, *n*	8
CTX‐M	4 (50%)
mecA/mecC	4 (50%)
Microorganisms identified exclusively by standard culture, *n*	24
*Candida albicans*, *n* (%)	8 (33.3%)
*Enterococcus faecalis*, *n* (%)	4 (16.7%)
*Stenotrophomonas maltophilia*, *n* (%)	2 (8.3%)
*Candida glabrata*, *n* (%)	1 (1.7%)
*Candida tropicalis*, *n* (%)	1 (1.7%)
*Candida* spp., *n* (%)	1 (1.7%)
*Candida kefyr*, *n* (%)	1 (1.7%)
*Candida parapsilosis*, *n* (%)	1 (1.7%)
*Aspergillus terreus* group, *n* (%)	1 (1.7%)
*Aspergillus fumigatus* group, *n* (%)	1 (1.7%)
*Trichosporon* spp., *n* (%)	1 (1.7%)
*Saccharomyces cerevisiae*, *n* (%)	1 (1.7%)
*Klebsiella pneumoniae*, *n* (%)	1 (1.7%)
Resistance genes identified by standard culture, *n*	2
CTX‐M	1 (50%)
mecA/mecC	1 (50%)

Abbreviation: VIM, Verona integron‐encoded metallo‐beta‐lactamase.

**FIGURE 1:**
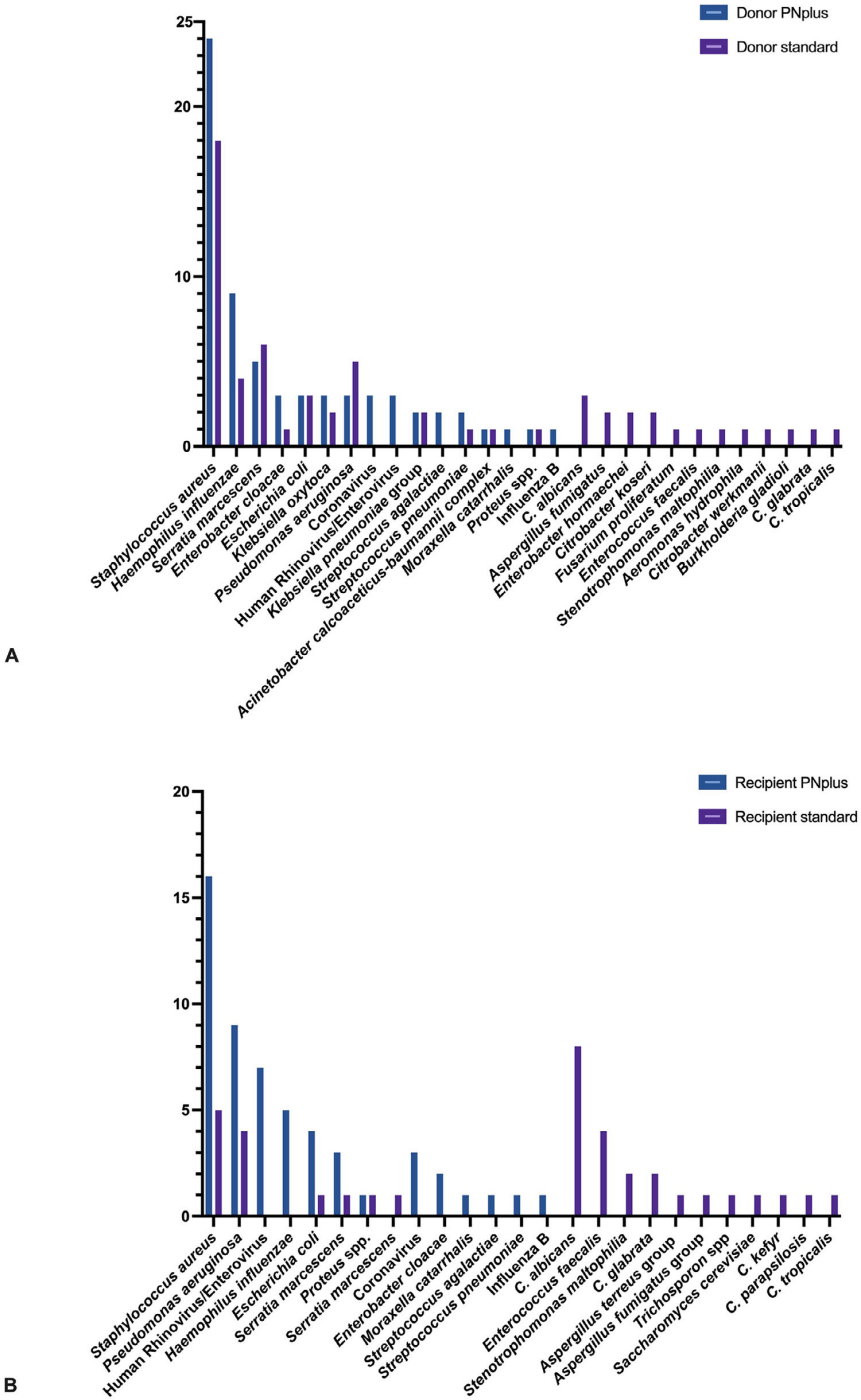
Microorganisms isolated on donor (A) and recipient (B) samples.

Figure 1A depicts the microorganisms isolated from the donor's sample, categorized according to the identifying instrument.

On the recipient's BAL sample, the PN*plus* detected at least one target in 32 (61.5%) patients, with an average time from sample acceptance in the microbiology laboratory to results availability of 157.7 min (SD 229.2). Conversely, SOC identified at least one microorganism in 25 patients (47.2%), with an average time from sample acceptance in the microbiology laboratory to definitive results of 4.9 days (SD 6). The microorganisms most frequently identified only by the PN*plus* were Human Rhinovirus/Enterovirus, *Haemophilus influenzae*, and Coronavirus, with seven, five, and three cases, respectively. Conversely, *C. albicans* and *Enterococcus faecalis* were the microorganisms most frequently identified only by SOC, with eight and four cases each. Regarding antimicrobial resistance genes, the PN*plus* detected eight targets: four CTX‐M and four mecA/mecC. Instead, SOC identified one CTX‐M and one mecA/mecC (Table 2).

Figure 1B depicts the microorganisms isolated from the recipient's sample, categorized according to the identifying instrument.

On the donor's BAL samples, the concordance between PN*plus* and SOC on bacterial species overall was substantial (Cohen's *κ* = 0.654), with solid results for both Gram‐negative (Cohen's *κ* = 0.729) and Gram‐positive bacteria (Cohen's *κ* = 0.654). Conversely, in the recipient's BAL samples, the agreement between PN*plus* and SOC regarding bacterial species overall was moderate (Cohen's *κ* = 0.365), with similar results for both Gram‐negative (Cohen's *κ* = 0.389) and Gram‐positive bacteria (Cohen's *κ* = 0.388). Concordance regarding antimicrobial resistance genes was almost absent in both donor and recipient samples (Table 3).

**TABLE 3:** Concordance between standard culture results and Pneumonia *plus* (PN*plus*) panel on donor samples and recipient samples.

	Donor		Recipient	
Concordance Gram‐positive				
No, *n* (%)	9 (17%)		11 (20.8%)	
Yes, *n* (%)	44 (83%)		42 (79.2%)	
Cohen's *κ*	0.654	*p < 0.001*	*0.388*	*p < 0.001*
Concordance Gram‐negative				
No, *n* (%)	7 (13.2%)		13 (24.5%)	
Yes, *n* (%)	46 (86.8%)		40 (75.5%)	
Cohen's *κ*	0.729	*p < 0.001*	0.389	*p < 0.001*
Concordance atypical pathogens				
No, *n* (%)	0		0	
Yes, *n* (%)	53 (100%)		53 (100%)	
Cohen's *κ*	*NA*		*NA*	
Concordance bacterial species overall				
No, *n* (%)	9 (17%)		17 (32.1%)	
Yes, *n* (%)	44 (83%)		36 (67.9%)	
Cohen's *κ*	0.654	*p < 0.001*	0.365	*p = 0.0013*
Concordance resistance genes				
No, *n* (%)	3 (5.7%)		6 (11.4%)	
Yes, *n* (%)	50 (94.3)		47 (88.7%)	
Cohen's *κ*	*NA*	*p = NA*	0.361	*p < 0.001*

When evaluated at a single target level, on the donor's BAL samples, the concordance was substantial for common and relevant pathogens such as *Staphylococcus aureus* (Cohen's *κ* = 0.689), *Streptococcus pneumoniae* (Cohen's *κ* = 0.658), *Pseudomonas aeruginosa* (Cohen's *κ* = 0.731), or *Serratia marcescens* (Cohen's *κ* = 0.899) (Table S2).

Instead, on the recipient's BAL samples, the concordance was moderate for *P. aeruginosa* (Cohen's *κ* = 0.571) or *S. marcescens* (Cohen's *κ* = 0.485), fair for *S. aureus* (Cohen's *κ* = 0.388) or *Escherichia coli* (Cohen's *κ* = 0.381), and absent for *H. influenzae* or *S. pneumoniae* (Cohen's *κ* = 0) (Table S3).

Regarding the impact of respiratory tract colonization or chronic infection, almost half of the recipients (24/53, 45.3%) had at least one microorganism isolated from respiratory tract samples in the 12 months preceding LuTx. As expected, most of these recipients (11/24, 45.3%) had cystic fibrosis and bronchiectasis as their reasons for transplant. *P. aeruginosa*, *Achromobacter* spp., and *S. aureus* were the bacteria more frequently encountered, with 14, five, and four isolates, respectively. Overall, 12.1% (4/33) of these isolates were *P. aeruginosa* with difficult‐to‐treat resistance (DTR), 9% (3/33) methicillin‐resistant *S. aureus* (MRSA), and 6.1% (2/33) expressed extended‐spectrum beta‐lactamases (ESBL) (Table S4).

The recurrence of these pathogens responsible for respiratory tract colonization or chronic infection was investigated in the recipient's BAL samples. *P. aeruginosa* recurred in half (7/14, 50%) of those with respiratory tract colonization or chronic infection, with higher recurrence rates according to PN*plus* (7/14, 50%) compared to SOC (4/14, 28.6%). Instead, *Achromobacter* spp. was not identified in any of the five recipients with a previous isolation (Table S5).

## 4 Discussion

This is the first prospective study assessing the concordance between PN*plus* and SOC in the unique setting of LuTx, evaluating these diagnostic approaches on both donor and recipient samples. Overall, considering the bacterial targets included in PN*plus*, the technique demonstrated substantial concordance on donor samples, particularly for significant pathogens such as *S. aureus*, *S. pneumoniae*, and *P. aeruginosa*. Instead, concordance was moderate in recipient samples, with acceptable results for *P. aeruginosa* or *S. aureus*, but no concordance for *H. influenzae* or *S. pneumoniae*. Considering resistance genes, the results were inadequate, with a higher number of identifications by PN*plus* compared to SOC in both donor and recipient samples. As expected, PN*plus* yielded faster results and detected a large number of viruses in both donor and recipient samples, which SOC cannot identify. On the contrary, fungi, including both pathogenic and non‐pathogenic species, were exclusively identified by SOC; however, this required a longer processing time. Finally, regarding pre‐LuTx respiratory tract colonization/chronic infection of the recipient, recurrence of the same isolate was frequent, particularly with pathogens such as *P. aeruginosa*, *S. aureus*, and *E. coli*, as indicated by PN*plus* results.

The concordance between PN*plus* and SOC in both LuTx donors and recipients had never been evaluated in previous studies. Nevertheless, the findings of Nguyen et al., who evaluated donor BAL samples using the PN*plus*, demonstrated a similarly high concordance between the syndromic panel and SOC for targets included in the panel, supporting the reliability of PN*plus* as a rapid tool for microbiological diagnostics in donor assessment [5].

In contrast, our results on recipient BAL at 72 h after LuTx showed a lower degree of concordance. No other investigations have assessed syndromic panels at this specific early post‐transplant time point; however, Hoover et al. reported high overall agreement (92%) in a cohort of LUTR where most BALs were obtained during routine surveillance rather than during suspected infection. The lower concordance in our study may be at least partly explained by the influence of recent surgical procedures, where microbial communities from different respiratory compartments are merged, and the concurrent use of PAP, which inevitably affects the sensitivity of SOC diagnostics [8].

The role of local epidemiology is further highlighted by the findings of Abdulqawi et al., who reported carriage of carbapenem‐resistant Gram‐negative bacteria in 20% of lung donors, predominantly *Acinetobacter baumannii*, and also described frequent discordance between PN*plus* and SOC for these isolates. By contrast, in our cohort, only one *Acinetobacter* spp. isolate was detected, without any carbapenem‐resistance gene, and agreement between methods was complete. This suggests that syndromic panels can be applied across various epidemiological settings, offering adaptable diagnostic support [9].

In line with prior observations, PN*plus* identified a high number of respiratory viruses in recipients, the clinical significance of which remains uncertain. Simon et al. also reported frequent viral detection by PN*plus* in LUTR with suspected respiratory infection. We speculate that the higher prevalence of viruses in early recipient samples may again reflect the blending of donor‐ and recipient‐derived respiratory tract microbiotas, with PN*plus* providing heightened sensitivity; however, further work is required to clarify the therapeutic implications of such findings [10].

As consistently reported in the literature, PNplus cannot detect fungi, an intrinsic limitation of the panel. Given the high‐risk of fungal disease in this population, SOC culture remains indispensable; nonetheless, the more indolent course of most fungal infections allows sufficient time for standard diagnostics, so this limitation should not preclude the role of PN*plus* in guiding PAP [11].

Finally, unlike previous studies, ours also evaluated resistance genes, where concordance between PN*plus* and SOC was absent or very low. PN*plus* frequently identified additional resistance determinants, likely reflecting its greater sensitivity in detecting traces of genetic material.

The principal limitation of this study lies in its single‐center design. This inevitably introduces a degree of selection bias, both in terms of patient characteristics, which largely reflect the traditional referral pool of our institution, and in terms of microbiological findings, which may be influenced by the center's prevailing microbial ecology. Nonetheless, it should be noted that our cohort comprised a variety of indications for LuTx, thereby ensuring a reasonable heterogeneity in the underlying conditions, even though suppurative lung diseases such as cystic fibrosis and bronchiectasis were relatively frequent and are typically associated with a more complex microbiological history. Furthermore, while the recipient population may reflect the center's specific case mix, the donor population is more representative at a national level, as organs were procured from intensive care units across Italy, each characterized by distinct microbial epidemiology. This broad geographic distribution of donors strengthens the generalizability of our results, particularly those regarding donor samples.

Among the study's strengths, its prospective nature and rigorous design should be emphasized. These features enabled a robust head‐to‐head comparison between PN*plus* and SOC in a specific and clinically challenging population. The systematic approach to sample collection and analysis reinforces the reliability of our findings and underscores their potential relevance for informing PAP strategies in LuTx.

The present study provides, for the first time, a systematic assessment of the performance of a syndromic molecular panel in both donor and early recipient BAL samples within the perioperative LuTx setting. Our findings indicate that the PN*plus* panel achieved substantial concordance with standard culture in donor samples, particularly for clinically relevant bacterial species, thereby supporting its role as a rapid adjunct to standard microbiology in donor evaluation. In contrast, concordance was markedly lower in early post‐transplant recipient samples, likely reflecting the unique challenges of this time window, including PAP and the mixture of donor‐ and recipient‐derived microbial communities. Taken together, these results suggest that PN*plus* may be most reliable when applied to donor samples for early tailoring of prophylaxis, while interpretation in the immediate post‐transplant recipient requires greater caution. The study therefore contributes to the evolving debate on how best to integrate syndromic molecular tools into perioperative strategies, complementing but not replacing conventional culture methods.

Several mechanisms may explain the differential performance of PNplus between donor and recipient samples. In donor lungs, microbial colonization is often dominated by a limited number of pathogens, facilitating concordance between culture and molecular testing. By contrast, in the early post‐transplant period, the airway environment is profoundly altered by surgical manipulation, immunosuppression, and broad‐spectrum PAP. These factors may reduce culture yield, while molecular assays remain capable of detecting residual genetic material from non‐viable organisms, thereby generating apparent discrepancies. Furthermore, the immediate postoperative state is characterized by the confluence of distinct microbial reservoirs: donor flora, recipient colonization, and perioperative environmental exposures, which may contribute to complex and transient detection patterns. The higher rate of viral identifications by PN*plus* could also reflect heightened molecular sensitivity in a context where the clinical significance of viral carriage remains uncertain. Finally, the discordance observed in resistance gene detection underscores intrinsic differences in methodology: molecular assays may identify low‐level genetic material not expressed phenotypically, whereas culture remains indispensable for confirming resistance and guiding targeted therapy.

Future studies should confirm the validity of our results using larger samples from different geographical areas and with diverse LuTx populations, as the prevalence of other pathogens may modify the outcomes. Moreover, the clinical and economic impact of PN*plus* integration in LuTx PAP adjustment should be assessed, particularly in terms of the medical significance of PN*plus* isolates and changes in antimicrobial consumption consequent to syndromic panel results.

## Author Contributions

A.L., G.R., and A.B. conceived the study. G.R., C.A., and A.Li. collected the clinical data. A.L. performed the statistical analyses. A.L. and G.R. wrote the first draft of the manuscript. All the authors reviewed the final version of the manuscript.

## Funding

This study was partially supported by IRCCS Ricerca Corrente 2025 funds.

## Conflicts of Interest

The authors declare no conflicts of interest.

## Supporting information




**Supporting Information File 1**: tid70186‐sup‐0002‐SuppMat.docx.

**Supporting Information File 2**: tid70186‐sup‐0001‐FigureS1.jpeg.

**Supporting Information File 3**: Visual Abstract.
