# Supplementary material for: Performance of the BioFire FilmArray Pneumonia Panel Plus Compared to Standard Microbiology in Lung Transplant Donor and Recipient Samples: A Prospective Cohort Study
Source: Transpl Infect Dis. 2026 Feb 20;28(3):e70186. doi: 10.1111/tid.70186 (PMC13262558; doi:10.1111/tid.70186)
Supplement: Supplementary file 1 — Supporting Information File 1: tid70186‐sup‐0002‐SuppMat.docx. [file TID-28-e70186-s003.docx]

**Supplementary table 1.** List of targets detected by Pneumonia *plus* (PN*plus*) panel according to the producer.

| **Gram-positive** | *Staphylococcus aureus* |
| --- | --- |
|  | *Streptococcus pneumoniae* |
|  | *Streptococcus pyogenes* |
|  | *Streptococcus agalactiae* |
|  |  |
| **Gram-negative** | *Escherichia coli* |
|  | *Enterobacter cloacae* |
|  | *Klebsiella aerogenes* |
|  | *Proteus* spp. |
|  | *Klebsiella pneumoniae* group |
|  | *Klebsiella oxytoca* |
|  | *Serratia marcescens* |
|  | *Moraxella catarrhalis* |
|  | *Pseudomonas aeruginosa* |
|  | *Acinetobacter calcoaceticus-baumannii* complex |
|  | *Legionella pneumophila* |
|  | *Haemophilus influenzae* |
|  |  |
| **Viruses** | Adenovirus |
|  | Coronavirus |
|  | Metapneumovirus |
|  | Rhinovirus/Enterovirus |
|  | Influenza A |
|  | Influenza B |
|  | Parainfluenza virus |
|  | RSV |
|  | MERS-CoV |
|  |  |
| **Atypical pathogens** | *Chlamydophila pneumoniae* |
|  | *Mycoplasma pneumoniae* |
|  |  |
| **Antibiotic resistance genes** | Carbapenemases: KPC, NDM, OXA-48 like, VIM, IMP |
|  | ESBL: CTX-M |
|  | Methicillin resistance: mecA/mecC and MREJ |

RSV, respiratory syncytial virus; MERS-CoV, Middle East Respiratory Syndrome Coronavirus; KPC, Klebsiella pneumoniae carbapenemase; NDM, New Delhi metallo-beta-lactamase; VIM, Verona integron-encoded metallo-beta-lactamase; IMP, imipenemase; MREJ, mec element right extremity junction.

**Supplementary table 2.** Concordance between Pneumonia *plus* (PN*plus*) panel and standard culture results for bacterial target and resistance genes on donor sample.

| **Target** | **PN*plus* positive** | | | **Concordance**  **(%)** | **Cohen’s *k*** | **P-value** |
| --- | --- | --- | --- | --- | --- | --- |
|  | **N** | **(PPV;NPV)** | **(Sens; Spec)** |  |  |  |
| *Staphylococcus aureus* | 24 | 0.71; 0.97 | 0.94; 0.80 | 84.9 | 0.689 | p<0.001 |
| *Streptococcus pneumoniae* | 2 | 0.5; 1 | 1; 0.98 | 98.1 | 0.658 | p<0.001 |
| *Streptococcus pyogenes* | 0 | NA; 1 | NA; 1 | 100 | NA | NA |
| *Streptococcus agalactiae* | 2 | 0; 1 | NA; 0.96 | 96.2 | 0.000 | NA |
| *Escherichia coli* | 3 | 1; 1 | 1; 1 | 100 | 1.000 | p<0.001 |
| *Enterobacter cloacae* | 3 | 0; 0.98 | 0; 0.94 | 92.5 | -0.029 | p=0.805 |
| *Klebsiella aerogenes* | 0 | NA; 1 | NA; 1 | 100 | NA | NA |
| *Proteus* spp. | 1 | 1; 1 | 1; 1 | 100.0 | 1.000 | p<0.001 |
| *Klebsiella pneumoniae* group | 2 | 1; 1 | 1; 1 | 100.0 | 1.000 | p<0.001 |
| *Klebsiella oxytoca* | 3 | 0.67; 1 | 1; 0.98 | 98.1 | 0.791 | p<0.001 |
| *Serratia marcescens* | 5 | 1; 0.98 | 0.83; 1 | 98.1 | 0.899 | p<0.001 |
| *Moraxella catarrhalis* | 1 | 0; 1 | NA; 0.98 | 98.1 | 0.000 | NA |
| *Pseudomonas aeruginosa* | 3 | 1; 0.96 | 0.6; 1 | 96.2 | 0.731 | p<0.001 |
| *Acinetobacter calcoaceticus-baumannii* complex | 1 | 1; 1 | 1; 1 | 100.0 | 1.000 | p<0.001 |
| *Legionella pneumophila* | 0 | NA; 1 | NA; 1 | 100 | NA | NA |
| *Haemophilus influenzae* | 9 | 0.44; 1 | 1; 0.90 | 90.6 | 0.571 | p<0.001 |
| *Chlamydophila pneumoniae* | 0 | NA; 1 | NA; 1 | 100 | NA | NA |
| *Mycoplasma pneumoniae* | 0 | NA; 1 | NA; 1 | 100 | NA | NA |
| VIM | 1 | 0; 1 | NA; 0.98 | 98.1 | 0.000 | NA |
| CTX-M | 1 | 0; 1 | NA; 0.98 | 98.1 | 0.000 | NA |
| mecA/mecC | 1 | 0; 1 | NA; 0.98 | 98.1 | 0.000 | NA |

**Supplementary table 3.** Concordance between the Pneumonia *plus* (PN*plus*) panel and standard culture results for each bacterial target on the recipient sample.

| **Target** | **PN*plus* positive** | | | **Concordance**  **(%)** | **Cohen’s *k*** | **P-value** |
| --- | --- | --- | --- | --- | --- | --- |
|  | **N** | **(PPV;NPV)** | **(Sens; Spec)** |  |  |  |
| *Staphylococcus aureus* | 16 | 0.31; 1 | 1; 0.77 | 79.2 | 0.388 | p<0.001 |
| *Streptococcus pneumoniae* | 1 | 0; 1 | NA; 0.98 | 98.1 | 0 | NA |
| *Streptococcus pyogenes* | 0 | NA; 1 | NA; 1 | 100 | NA | NA |
| *Streptococcus agalactiae* | 1 | 0; 1 | NA; 0.98 | 98.1 | 0 | NA |
| *Escherichia coli* | 4 | 0.25; 1 | 1; 0.94 | 94.3 | 0.381 | p<0.001 |
| *Enterobacter cloacae* | 2 | 0; 1 | NA; 0.96 | 96.2 | 0 | NA |
| *Klebsiella aerogenes* | 0 | NA; 1 | NA; 1 | 100 | NA | NA |
| *Proteus* spp. | 1 | 1; 1 | 1; 1 | 100.0 | 1 | p<0.001 |
| *Klebsiella pneumoniae* group | 0 | NA; 0.98 | 0; 1 | 98.1 | 0 | NA |
| *Klebsiella oxytoca* | 0 | NA; 1 | NA; 1 | 100 | NA | NA |
| *Serratia marcescens* | 3 | 0.33; 1 | 1; 0.96 | 96.2 | 0.485 | p<0.001 |
| *Moraxella catarrhalis* | 1 | 0; 1 | NA; 0.98 | 98.1 | 0 | NA |
| *Pseudomonas aeruginosa* | 9 | 0.44; 1 | 1; 0.90 | 90.6 | 0.571 | p<0.001 |
| *Acinetobacter calcoaceticus-baumannii* complex | 0 | NA; 1 | NA; 1 | 100 | NA | NA |
| *Legionella pneumophila* | 0 | NA; 1 | NA; 1 | 100 | NA | NA |
| *Haemophilus influenzae* | 5 | 0; 1 | NA; 0.91 | 90.6 | 0 | NA |
| *Chlamydophila pneumoniae* | 0 | NA; 1 | NA; 1 | 100 | NA | NA |
| *Mycoplasma pneumoniae* | 0 | NA; 1 | NA; 1 | 100 | NA | NA |
| CTX-M | 4 | 0.25; 1 | 1; 0.94 | 94.3 | 0.381 | p<0.001 |
| mecA/mecC | 5 | 0.25; 1 | 1; 0.94 | 92.5 | 0.312 | p=0.002 |

**Supplementary table 4.** Respiratory tract colonisation or chronic infection within the 12 months prior to transplantation.

| **N** | **53** |
| --- | --- |
| **Respiratory tract colonization/chronic infection** | |
| Respiratory tract colonized/chronically infected, n (%) | 24 (45.3%) |
| Colonization/chronic infection assessment before transplantation (>=1 assessment), days (Median and IQR)° | First assessment  108.5 (181)  Last assessment  54.5 (160) |
| Colonization/chronic infection assessment before transplantation (>=2 assessment), days (Median and IQR)° | First assessment  108.5 (88)  Last assessment  41 (55) |
| Respiratory tract colonized/chronically infected according to transplant indication^§^ |  |
| Cystic fibrosis and bronchiectasis, n (%) | 11 (45.8%) |
| ILD, n (%) | 1 (4.2%) |
| Connective tissue disease, n (%) | 1 (4.2%) |
| COPD, n (%) | 3 (12.5%) |
| Lymphangioleiomyomatosis and sarcoidosis, n (%) | 1 (4.2%) |
| Other, n (%) | 6 (25%) |
|  |  |
| Microbiological isolates* | 33 |
| *Pseudomonas aeruginosa*, n (%) | 14 (42.4%) |
| *Achromobacter* spp., n (%) | 5 (15.2%) |
| *Staphylococcus aureus*, n (%) | 4 (12.1%) |
| *Escherichia coli*, n (%) | 3 (9.1%) |
| *Candida* spp., n (%) | 2 (6.1%) |
| *Haemophilus influenzae*, n (%) | 1 (3%) |
| *Klebsiella* spp., n (%) | 1 (3%) |
| *Serratia marcescens*, n (%) | 1 (3%) |
| *Stenotrophomonas maltophilia*, n (%) | 1 (3%) |
| *Aspergillus* spp., n (%) | 1 (3%) |
|  |  |
| Resistance mechanism/profile of isolates |  |
| MRSA, n (%) | 3 (9%) |
| CTX-M, n (%) | 2 (6.1%) |
| KPC, n (%) | 0 |
| NDM, n (%) | 0 |
| VIM, n (%) | 0 |
| IMP, n (%) | 0 |
| OXA-48, n (%) | 0 |
| *Aspergillus* spp. azole-resistant, n (%) | 0 |
| *Candida* spp. azole-resistant, n (%) | 0 |
| *P. aeruginosa* DTR, n (%) | 4 (12.1%) |
| *P. aeruginosa* XDR, n (%) | 0 |

MRSA, methicillin-resistant *Staphylococcus aureus*; KPC, Klebsiella pneumoniae carbapenemase; NDM, New Delhi metallo-beta-lactamase; VIM, Verona integron-encoded metallo-beta-lactamase; IMP, imipenemase; OXA-48, oxacillinase-48; DTR, difficult-to-treat resistance; XDR, extensively drug-resistant.

*Assessment of respiratory tract colonisation/chronic infection could have been performed more than once in each patient in the 12 months before transplantation.

°More than one indication possible for each patient.

**Supplementary table 5.** Recurrence of microbiological results in patients with respiratory tract colonisation/chronic infection before transplantation in recipient samples, based on the Pneumonia *Plus* (PN*plus*) panel and standard culture outcomes.

| **Microorganism or resistance mechanism/profile** | **Recipients with solation before transplantation,**  **n** | **Recipient with recurrence after transplantation** | | |
| --- | --- | --- | --- | --- |
|  |  | **Overall,**  **n (%)** | **PN*plus*,**  **n (%)** | **Standard culture, n (%)** |
| Microorganisms |  |  |  |  |
| *Pseudomonas aeruginosa* | 14 | 7 (50%) | 7 (50%) | 4 (28.6%) |
| *Achromobacter* spp. | 5 | 0 | 0 | 0 |
| *Staphylococcus aureus* | 4 | 2 (50%) | 2 (50%) | 1 (25%) |
| *Escherichia coli* | 3 | 2 (66.6%) | 2 (66.6%) | 1 (33.3%) |
| *Candida* spp. | 2 | 0 | 0 | 0 |
| *Haemophilus influenzae* | 1 | 0 | 0 | 0 |
| *Klebsiella* spp. (non-pneumoniae) | 1 | 0 | 0 | 0 |
| *Serratia marcescens* | 1 | 0 | 0 | 0 |
| *Stenotrophomonas maltophilia* | 1 | 1 (100%) | 0 | 1 (100%) |
| *Aspergillus* spp. | 1 | 0 | 0 | 0 |
|  |  |  |  |  |
| Resistance mechanisms/profile |  |  |  |  |
| mecA/mecC | 3 | 1 (33%) | 1 (33%) | 0 |
| CTX-M | 2 | 2 (100%) | 2 (100%) | 1 (50%) |
| *P. aeruginosa* DTR | 4 | 2 (50%) | 0 | 2 (50%) |

MRSA, methicillin-resistant *Staphylococcus aureus*; DTR, difficult-to-treat resistance.

**Supplementary figure 1.** Study flowchart.
