# Supplementary figures and images for: Performance of the BioFire FilmArray Pneumonia Panel Plus Compared to Standard Microbiology in Lung Transplant Donor and Recipient Samples: A Prospective Cohort Study
Source: Transpl Infect Dis. 2026 Feb 20;28(3):e70186. doi: 10.1111/tid.70186 (PMC13262558; doi:10.1111/tid.70186)

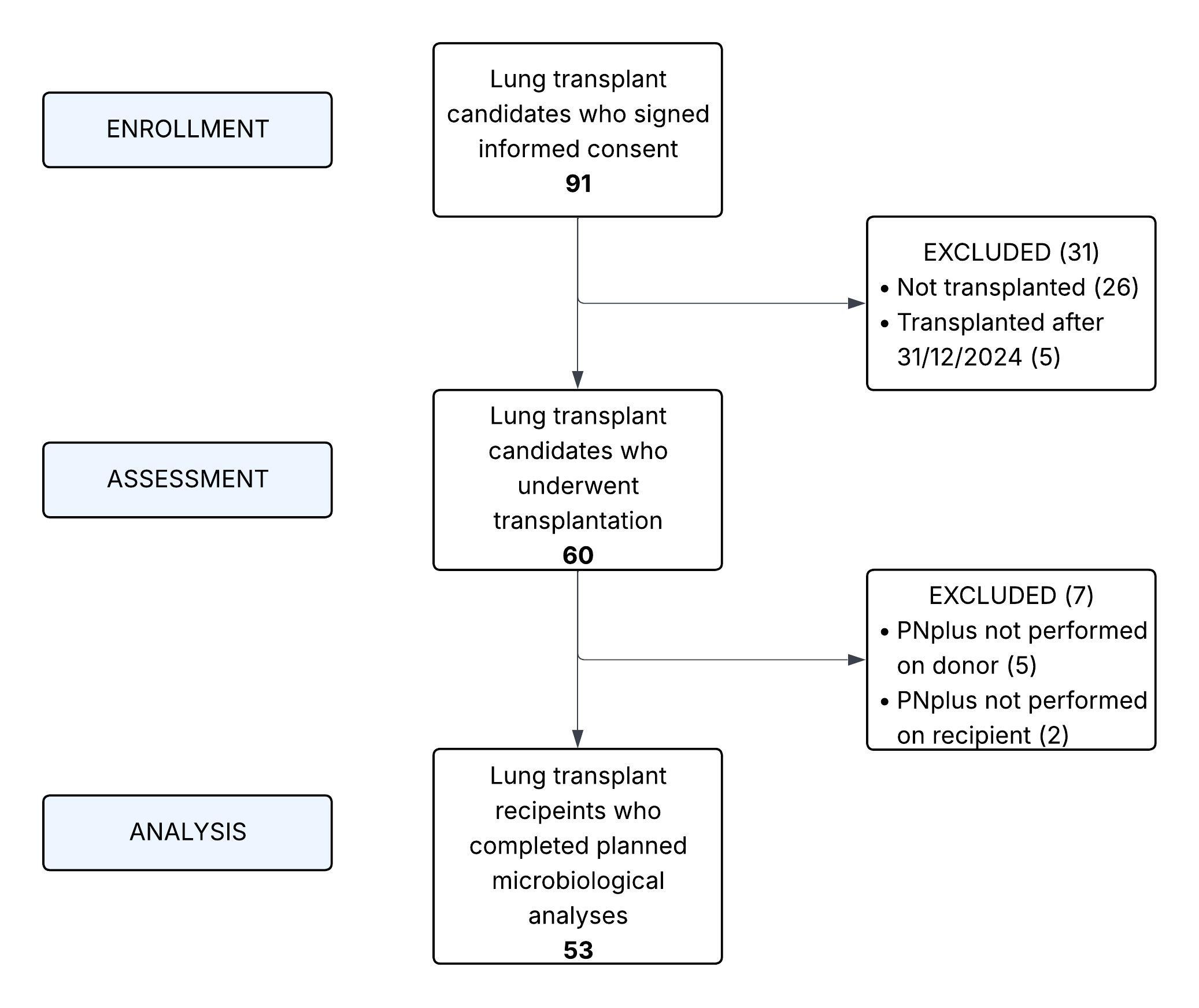

Supplement: Supplementary file 2 — Supporting Information File 2: tid70186‐sup‐0001‐FigureS1.jpeg. [file TID-28-e70186-s002.jpeg]
